# General Movements in Infants with Neurological Risk: Associations with Motor Development and Referral Patterns for Brain Magnetic Resonance Imaging

**DOI:** 10.3390/children12050590

**Published:** 2025-04-30

**Authors:** María Eugenia Serrano-Gómez, Núria Massó-Ortigosa, Adriana Lucía Castellanos-Garrido, Eduardo Acuña De La Rosa, Víctor Mauricio García-Barriga, Adriana López-Dóriga, Małgorzata Domagalska-Szopa, Andrzej Szopa, Magdalena Hagner-Derengowska, Myriam Guerra-Balic

**Affiliations:** 1 Faculty of Psychology, Education and Sport Sciences, Blanquerna, University Ramon Llull, 08022 Barcelona, Spain; miriamelisagb@blanquerna.url.edu; 2 Departamento del Movimiento Corporal Humano, Universidad Nacional de Colombia, Bogotá 111321, Colombia; 3 Faculty of Health Sciences, Blanquerna, University Ramon Llull, 08022 Barcelona, Spain; nuriamo@blanquerna.url.edu; 4 Facultad de Enfermería y Rehabilitación, Universidad de La Sabana, Chía 50260, Cundinamarca, Colombia; adrianacasga@unisabana.edu.co; 5 Hospital Universitario de La Samaritana, Bogotá 110311, Colombia; eduardo.acuna1@unisabana.edu.co (E.A.D.L.R.); neonatos.lider@hus.org.co (V.M.G.-B.); 6 Nennisiwok AI Lab, 08024 Barcelona, Spain; adriana.ldoriga@gmail.com; 7 Department of Developmental Age Physiotherapy, Medical University of Silesia, 40-752 Katowice, Poland; mdomagalska@sum.edu.pl; 8 Department of Physiotherapy, Medical University of Silesia, 40-752 Katowice, Poland; aszopa@sum.edu.pl; 9 Rehabilitation and Medical Center Neuromed SC, 40-698 Katowice, Poland; 10 Faculty of Earth Sciences and Spatial Management, University Nicolaus Copernicus, 87-100 Torun, Poland; madzixhag@wp.pl

**Keywords:** neurological risk, general movements, motor development, magnetic resonance imaging, brain injuries, physical therapy specialty

## Abstract

The main goal of this study was to determine the associations between the quality of presentation of GM, motor development, and brain integrity as seen through magnetic resonance imaging. Methods: This is an observational, descriptive, and association study; information derived from it was used to analyze associations between the following variables: Writhing Movements, Fidgety Movements, motor development, and brain integrity. With a confidence level of 95% and an estimation error of 5%, the sample was comprised of 60 children under 5 months old with any neurological risk criteria; these children were either hospitalized in the Neonatal Intensive Care Unit (NICU) or attending the Kangaroo Mother Care Program (KMCP) at the University Hospital of La Samaritana (UHS), Colombia. The data were analyzed using Fisher’s exact test. Results: Over 90% of children with Absent or Sporadic Fidgety Movements had either abnormal or suspicious motor development. We observed a trend of association between the absence of Fidget Movements and alterations in White Matter. Conclusions: Quality of presentation of General Movements is associated with motor development and alterations of brain tissue at an early age, primarily in the White Matter; it is important for early prediction of neurological risk in infants.

## 1. Introduction

Neurological risk is often associated with brain injuries that occur during the pre or perinatal period. One of the primary outcomes of neurological risk is Cerebral Palsy (CP), a condition considered the leading cause of disability of physical-motor origin [1].

The World Health Organization estimates a CP prevalence of 1.5% in Colombia, affecting approximately 719,000 individuals [2]. The clinical signs of CP show from a very early age, but the diagnosis is generally given some time during the first two years of the child [3]; CP is often diagnosed within the first two years, though this window may be too broad to capitalize on early neuroplasticity [4].

Diverging from neuroimaging methods, Prechtl developed the General Movements (GM) method, an observational method that has shown a high predictive value for the diagnosis of neurological dysfunctions such as CP; it is a valid and reliable diagnostic tool that requires training for whoever applies it and aids on starting intervention oriented towards the enhancement of neural processes associated to brain plasticity [5,6,7,8,9].

GM can be classified as normal or abnormal; within the normal movements, one can find the following: Writhing Movements from birth up to the eight weeks post-term; these have an ellipsoid motion, a small to moderate amplitude, and a low to moderate speed. These include rotational movements that are varied, unpredictable, and fluid; their recession coincides with the gradual appearance of Fidgety Movements [6]. These latter ones can be found between the ninth and twentieth weeks post-term and are continuous, have a small amplitude, moderate speed, and variable acceleration; they can be found on the neck, torso, and limbs, and their absence is a flag strongly associated with CP [6,10].

When Writhing Movements appear as monotonous, not fluid, without any sequence nor rotational movement, they are referred to as a Poor Repertoire (PR), and it can precede the emergence of another kind of movement—one that has always abnormal—called Cramped Synchronized Movements (CSM). CSM can be seen as sudden and almost simultaneous whole-body contractions and subsequent relaxation; they have a period of continuous muscular activation followed by a relaxation period. When these are seen constantly, they are highly suggestive of the development of spastic CP [6].

Another set of movements considered abnormal is Chaotic Movements (CM); these are uncommon and are seen in the limbs; they are disorganized, of great amplitude, harsh, abrupt, shaky, and lack any fluidity [6].

Despite the strong predictive value of GM assessment, there is limited research linking GM quality to specific brain alterations. This study addresses that gap by investigating the association between GM and magnetic resonance imaging (MRI) findings in a Colombian cohort of infants at neurological risk. By exploring how GM abnormalities correlate with brain integrity, we aim to improve early identification strategies, particularly in resource-limited settings where neuroimaging access is restricted. Understanding the relationship between GM quality and MRI findings will be useful for patient management, especially in situations where neuroimaging is difficult or unaffordable, as is the case for a significant number of Colombian families.

## 2. Materials and Methods

This article deals with a descriptive and association study [11]. The sample was comprised of 60 children under 5 months post-term, whether preterm or full-term born. All of these children were either hospitalized in the NICU or attending the KMCP at the UHS between October 2022 and November 2023. Given that each child’s assessment required following up on their development up to the 17–20th-week post-term, the observation period extended up to March 2024.

### 2.1. Sample Size

To determine sample size, both a finite population and the main qualitative research variable were taken into account; after establishing a confidence level of 95% (Z-score: 1.96), there was an estimation error of 5% and a 10% chance of the event (association between extreme prematurity and brain damage [12]), and q = 90%. Based on this, and taking an average population size of 108 children within the inclusion criteria assisting the UHS’ KMCP, a sample size of 60 children was decided upon. The algebraic expression to calculate it is as follows [13]:

(1) $$n=\frac{N\times Z_{\alpha }^{2}\times p\times q}{e^{2}\times (N-1)+Z_{\alpha }^{2}\times p\times q}$$

Any child who met at least one of the following criteria associated with neurological risk was included in the study: past perinatal asphyxia, low weight at birth (under 1500 g), gestational age under 32 weeks, abnormal results in brain echography (grade III or IV hemorrhages), abnormal motor development around three months post-term (as determined through the Alberta Infant Motor Scale (AIMS)).

A descriptive analysis of all variables was performed; participants were excluded due to their deaths or multiple hospitalizations and were not taken into account for the statistical analysis. If parents denied the child’s participation at any point in time, they were also rejected. Descriptive statistics methods were used to characterize the population. In order to understand the associations between the main variables of the study, a bivariate association analysis was carried out, with the variables categorized within dichotomies as follows: Writhing Movements: Normal (N) and Poor Repertoire (PR); Fidgety Movements: “Normal” (F++) and “Absent” as a category that included Absent Fidgety Movements (F−) and Sporadic Fidgety Movements (F+/−); motor development: Without abnormalities (Normal and Suspicious) and Abnormal (Abnormal); brain integrity: Without alteration and With alteration. Based on the sample size, the bivariate analyses were conducted through Fisher’s exact test; associations with a *p*-value < 0.05 (95% confidence) were deemed significant. Analyses were carried out with the R 4.1.2 software.

### 2.2. Measurement Instruments

The following instruments were used for measurements: AIMS, GM Assessment Test, and brain magnetic resonance imaging (MRI).

### 2.3. Procedure

Each child’s parents signed the informed consent form on the first day of assessment. Two or three video recordings were scheduled depending on the child’s age at the moment of joining the study. Children who were able to join the study prior to their seventh week post-term underwent three assessment moments: one during their Writhing stage and two during the Fidgety stage between the twelfth and sixteenth weeks post-term and the seventeenth and twentieth weeks post-term, respectively. Children who were able to join the study in the Fidgety stage underwent two assessment moments between the twelfth and sixteenth weeks post-term and the seventeenth and twentieth weeks post-term, respectively.

The Writhing stage assessment had the goal of identifying between Normal and Abnormal (PR, CSM or CM) Writhing Movements. The Writhing stage assessment was performed after they were released from the hospital, during a KMCP consultation or at home.

The Fidgety stage assessment had the goal of identifying the quality of presentation (Normal Fidgety Movements, Absent Fidgety Movements, Abnormal Fidgety Movements), and for both moments, the videos were recorded during a KMCP consultation or at home.

Each GM video was observed and analyzed blind and independently by two researchers officially trained in the GM Trust Method; each of them registered their observations in a Google Form sent by the main researcher, who took all of the videos. After the results were sent in, the main researcher—who is also trained in the GM Trust Method—checked said results and worked through the discrepancies, filling in the role of the third evaluator. The risk of bias from the evaluators was minimized by showing them the video recordings without them having any knowledge of the MRI results. There were six children with discrepancies in their assessments from evaluators one and two; among these videos, the main researcher regarded four of them as having a higher difficulty level for analysis, the reason for which she sent them to Italian physiotherapist Natascia Bertoncelli, official instructor of the “General Movements Trust”, in order to work through their ambiguity.

Motor development was assessed by the main researcher through the use of the AIMS on the same day as the second Fidgety Movements assessment.

Referral for an MRI was based on the routine check-ups performed by the children’s pediatricians at the KMCP and by the main researcher of the study; they all reported the findings from the check-ups in the medical history, which was the supporting evidence to refer children for a brain MRI scan.

### 2.4. Ethical Considerations

This study took into account both the recommendations found in the Helsinki Declaration [14] and the Colombian regulations established by Resolution 8430 of 1993 from the Health Ministry [15].

Recruiting children into the study was performed with the will and informed consent of the parents. Video recordings were encrypted and labeled with alphanumeric codes. All participants benefited from the investigation because its results were all included in their medical history; in those cases where motor development deviations were found, the main researcher instructed the parents to perform early stimulation activities at home and went through the necessary protocols for them to access the KMCP’s physiotherapy services.

This research was part of a wider research project from the La Sabana University in Colombia called “Cerebral Palsy, General Movements, Motor Development in Infants and Management Strategies for Families”. Because of this, it had the approval of the Ethics and Research Committees at the La Sabana University (Code 022, 20 November 2020) and the La Samaritana University Hospital (Code 092022, 29 September 2022), as well as the approval by the Ethics and Research Commission from the Ramon Llull Blanquerna University (Code 1920010D, 12 June 2020).

## 3. Results

### 3.1. Sample Characterization

The current research had 59 children with neurological risk as a sample, all of whom fit the criteria set by its protocol; nine children were under circumstances related to withdrawal criteria, which is why the study closed with a sample of 50 children who had completed the proposed assessments. Informed consent had been received from the parents of all the withdrawn children. The reasons for withdrawal from the study were as follows: four due to refusal from the parents to continue with their participation; two due to their death around their first month post-term; one due to multiple hospitalizations, which made follow-ups impossible; one due to ending up under the care of the Colombian Family Welfare Institute, which made it so they would have to go through multiple, long drawn legal procedures to obtain permission for them to continue participating in it; and the last due to having a confirmed diagnosis of a genetic illness during the second month post-term.

Thirty-three children (66%) were included right from their stay at the NICU, while seventeen children (34%) started through their KMCP regular check-ups at the La Samaritana Hospital. Out of the entirety of the sample, 45 children (90%) were born preterm. All of the samples came from the twelve municipalities of La Sabana de Bogotá, with a higher number coming from Zipaquirá (n = 20) and Ubaté (n = 9).

According to the inclusion criteria for the study, both term-born and preterm children were included, with gestational ages between 25 and 40 weeks, all of whom had at least one criterion associated with neurological risk. The sample had predominantly children of male sex (56%) and “very preterm” infants (58%). There was a predominance of children who presented episodes of bronchopulmonary dysplasia (70%) and respiratory distress at birth (98%) (Table 1).

### 3.2. Associations Between General Movements and Brain Integrity as Seen Through Magnetic Resonance Imaging

Out of the 50 children included in the sample by the end of the study, 35 children (70%) were assessed to have normal motor development, 5 (10%) had suspicious motor development, and 10 (20%) had abnormal motor development.

**Figure 1:**
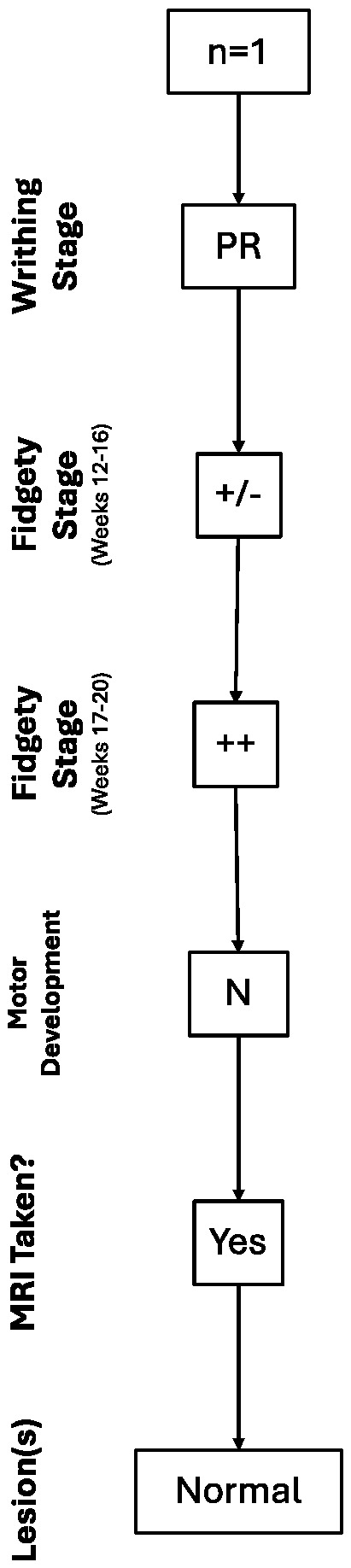
Trajectory of the case with a normal brain MRI result.

Out of the 35 children with normal motor development, only one of them underwent an MRI scan as they had a history of PR and, for the second assessment moment (Fidgety stage: 12–16 weeks post-term), had Sporadic Fidgety associated with a monotonous heel-tibia kicking pattern and abnormal tongue movements. Those were taken into account for the child’s referral for a brain MRI, even after having observed Normal Fidgety during the third assessment moment (Fidgety stage: 17–20 weeks post-term). In this case, the brain MRI results were found within normal parameters; thus, no brain lesions or alterations were found (Figure 1).

**Figure 2:**
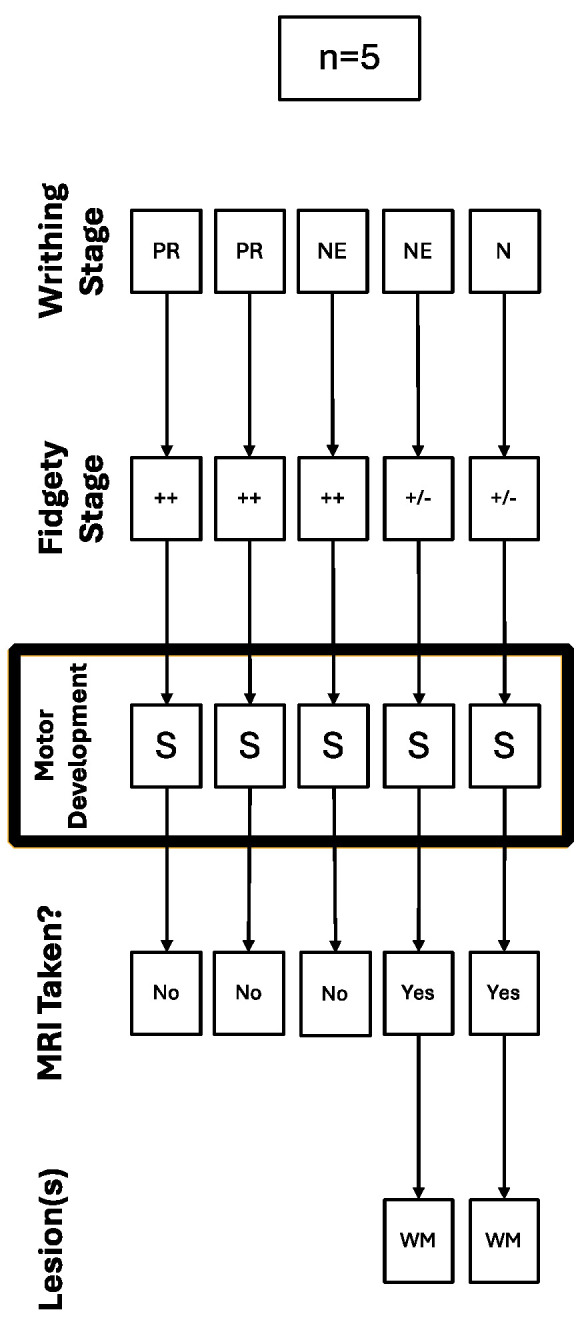
Trajectory of the cases with suspicious motor development referred for a brain MRI.

Out of the five children deemed to have a suspicious (S) motor development, three had Normal Fidgety during the Fidgety stage, and because of that, they were not referred for a brain MRI; this was performed even though two of them (67%) had a history of PR for the Writhing stage. The other two children deemed to have a suspicious (S) motor development had persistent Sporadic Fidgety and were referred for an MRI with abnormal results, finding lesions in the WM for both cases (Figure 2).

**Figure 3:**
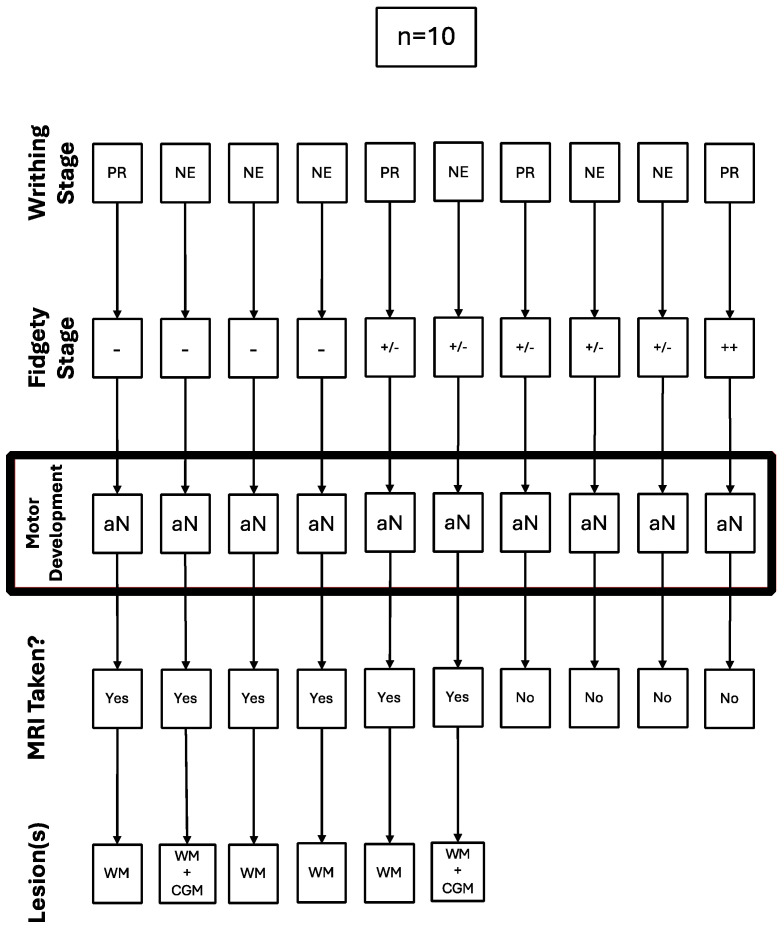
Trajectory of the cases with abnormal motor development referred for a brain MRI.

Out of the children with abnormal motor development (n = 10), four (40%) had Absent Fidgety, five (50%) had Sporadic Fidgety, and one (10%) had Normal Fidgety; the latter was not referred for an MRI (Figure 3).

All of the five children with Sporadic Fidgety and abnormal motor development were ordered an MRI, but only two of them had the chance to undergo one, both of whom had results showing alterations on the WM, and one of them had alterations on the Cortical Gray Matter (CGM) (Figure 3).

All of the four children with Absent Fidgety and abnormal motor development were ordered and underwent a brain MRI, with 100% of their results showing alterations on the WM and one of them (25%) showing additional alterations on the CGM (Figure 3).

**Figure 4:**
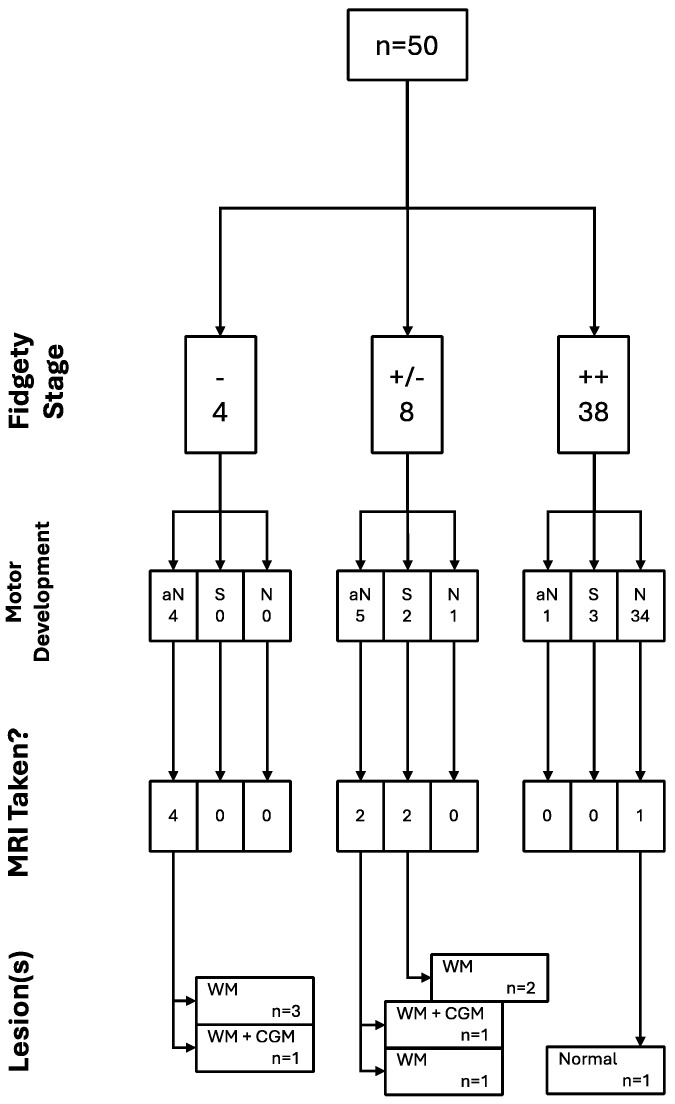
Relational analysis between Fidgety Movements and brain integrity as seen through MRI (n = 9).

**Figure 5:**
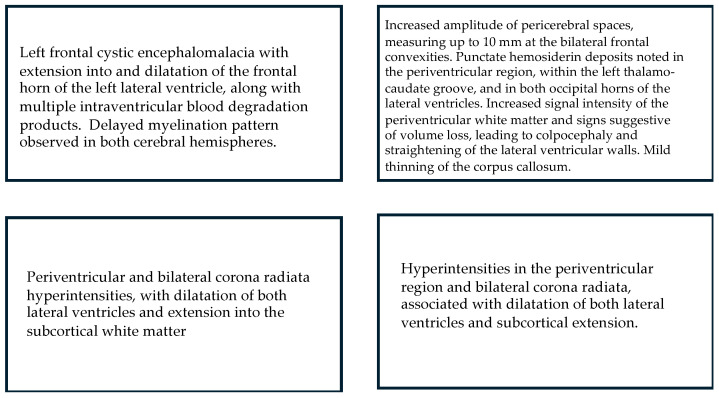
MRI: Main results for children who displayed Absent Fidgety.

**Figure 6:**
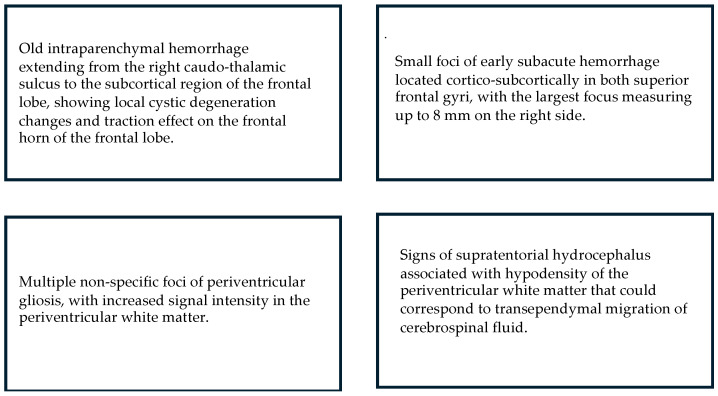
MRI: Main results for children who displayed Sporadic Fidgety.

Out of the sample in the current research, nine children total were able to undergo MRI scans; one of them had Sporadic Fidgety during the first Fidgety stage assessment and Normal Fidgety during the second one; the MRI results concluded on normalcy of the brain tissue and encephalic regions. The other eight children with MRI results had persistent Absent Fidgety or Sporadic Fidgety; out of them, 100% showed WM lesions, and two had an associated lesion on the CGM (Figure 4, Figure 5 and Figure 6).

**Figure 7:**
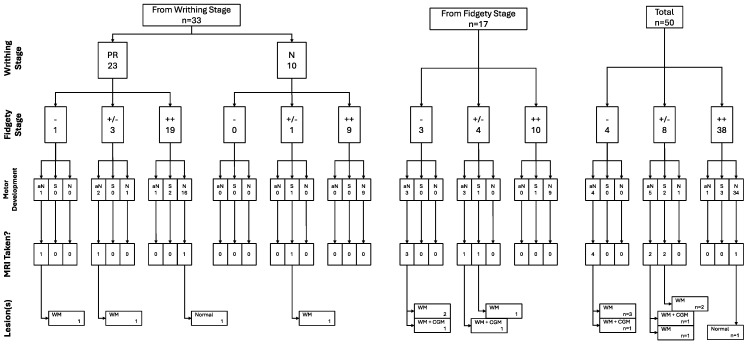
GM, motor development, and brain integrity as seen through MRI trajectories (n = 50).

All of the trajectories and their associations throughout the assessment window for all children included in the study can be found in the following figure (Figure 7).

### 3.3. Associations Between Writhing Movements and Fidgety Movements

Although this study had a sample size of 50 children, only 33 of them were included in it from their stay at the NICU, having had neurological risk identified during their first weeks of being born; because of this, the associations between Writhing Movements and Fidgety Movements was performed with a sample of 33 children.

According to the following statistical analysis, there is no significant association between the Writhing Movements and Fidgety Movements variables (*p*-value = 0.171).

### 3.4. Associations Between General Movements and Motor Development

According to the statistical analysis, there is no significant association between the variables Writhing Movements and motor development (*p*-value = 0.298).

On the other hand, the relational analysis between Fidgety Movements and motor development included all 50 children as samples, taking into account both children taken in through the first and the second routes.

According to the statistical analysis for this, there is a significant association between the variables Fidgety Movements and motor development (*p*-value < 0.001) (Table 2).

## 4. Discussion

This study aims to contribute to the early detection of neurological risk through the use of validated and reliable measurements. To date, no studies in Colombia have explored the relationship between General Movements (GM) and motor development or brain integrity. Furthermore, this is the first research conducted on infants at neurological risk in the Sabana de Bogotá region.

### 4.1. On the Association Between Motor Development and the Quality of General Movements

Our study did not find a significant association between the quality of Writhing Movements and motor development. However, statistical analysis revealed a significant association between Fidgety Movements and motor development.

Consistent with our findings, Yildirim et al. [16] reported a strong correlation between GM and motor development assessed via the AIMS (Kappa coefficient: 0.804; *p*-value = 0.001) during the Fidgety stage. They also identified a correlation during the Writhing stage. Their study concluded that combining GM assessments with motor development evaluations (AIMS) may enhance clinical decision-making. However, it is important to note that while GM assessment is predictive of neurodevelopmental outcomes, it is not diagnostic, particularly in small observational samples.

Snider et al. examined the concurrent validity of GM assessments with traditional tests such as the Test of Infant Motor Performance (TIMP) and AIMS. Their findings suggested low correlation indices, indicating that GM assessments represent a distinct construct different from traditional measures of postural and behavioral responses. They also found a strong relationship between TIMP and AIMS, likely due to shared underlying constructs [17].

### 4.2. On the Association Between Writhing Movements and Fidgety Movements

Our analysis did not reveal a significant association between Writhing Movements and Fidgety Movements. Notably, during the Writhing stage, no child exhibited CSM or CM. However, a high percentage (70%) displayed PR, and of these, 83% were scored as Normal during the Fidgety stage.

These findings align with Van Iersel et al., who studied GM trajectories in children with perinatal asphyxia. Their research found that 75% of children exhibited Abnormal Writhing Movements, but 31% of them normalized their repertoire during the Fidgety stage. This suggests a transient dysfunction in the nervous system of children at neurological risk and underscores the intrinsic recovery potential through cerebral plasticity mechanisms [18].

While our study did not include children with CSM, previous research has established a strong correlation between CSM and CP, with sensitivity indices reaching 100% [19,20].

### 4.3. On the Associations Between the Quality of GM and Brain Integrity

#### 4.3.1. White Matter

Certainly, WM lesions are among the most commonly reported findings in studies investigating the relationship between brain integrity and GM anomalies.

We failed to find an association between the absence of Fidgety Movements and alterations in WM. This may be due to the small sample size in our study; however, associations between those variables have been reported by Want et al. [21], Harpster et al. [22], and Spittle et al. [23]. Additionally, Peyton et al. [24,25] described a relationship between WM alterations and Aberrant Fidgety Movements, including Absent Fidgety, Abnormal or Exaggerated Fidgety (AF), and Sporadic Fidgety. The GM Trust recommends categorizing Sporadic Fidgety observed between 10 and 15 weeks post-term as Absent Fidgety, suggesting that the strongest correlation exists between WM alterations and absence of Fidgety Movements. Accordingly, our study included Sporadic Fidgety within the Absent Fidgety category for statistical analysis.

No associations were found between Writhing Movements and WM lesions, nor did our study observe trends supporting such a link. However, Maeda et al. [26] reported that WM deficiencies correlate with Abnormal Writhing Movements without distinguishing between PR, CM, or CSM.

In general, WM lesions have been characterized in previous studies using markers such as signal anomalies, decrease in the volume of the Periventricular WM, ventricular dilatation, decreased Bifrontal and Biparietal Diameters, cystic degeneration, thinning of the Corpus Callosum, delays in myelination, specific impairment of the Superior and Inferior Longitudinal Fasciculus, the Fronto-Occipital Fasciculus, the Anterior and Posterior Limb of the Internal Capsule, the Corona Radiata, and the Optic Radiation. In our study, WM alterations were estimated through the following markers: increase in WM signals/Periventricular and Corona Radiata Hyperintensity, Colpocephaly/dilation of the Lateral Ventricles, rectifying the walls of Lateral Ventricles, thinning of the Corpus Callosum, and periventricular gliosis.

The consistent association between increased Periventricular WM signal and abnormal GM presentation supports the hypothesis that GM abnormalities may reflect disruptions in cortical–subcortical connections interacting with Periventricular WM [18].

WM plays a key role in integrating neurological functions via myelinated axons, forming a network that connects various regions of the central nervous system. Damage to Periventricular WM is frequently observed in preterm children and may manifest as cystic lesions, myelination delays, volume loss, and corpus callosum thinning. These alterations are strongly associated with Spastic CP and may co-occur with cognitive dysfunction and learning difficulties [27,28,29].

#### 4.3.2. Cortical and Deep Gray Matter

Two of the children who underwent MRI scans for our study appeared to have alterations on the CGM; these children were taken into the study after the Writhing stage, which is why there are no registers of their assessments during that stage; one of them has a history of Absent Fidgety, and the other presented Sporadic Fidgety. Unlike ours, other studies carried out by Olsen et al. [30] and Maeda et al. [26] found associations between CGM and Abnormal Writhing Movements, including PR, CM, and CSM; these findings were not identified in our study.

Without including them within Abnormal Writhing Movements, Ferrari et al. [31] isolated CSM to study them and found that these have a definitive association with Basal Nuclei (BN) lesions, along with abnormal patterns for the Thalamus. In their own study, Harpster et al. [22] found a correlation between Deep Gray Matter (DGM) and the absence of Fidgety Movements. Our study did not find any DGM lesions in the MRI results.

It is known that lesions in the CGM are associated with Spastic PC, that those in the DGM are primarily seen as Dyskinetic CP, and those found around the Germinal Matrix with BN affectations have a strong association with adverse prognosis for walking [32].

Lesions in the CGM are not very common in preterm children; however, there is a sizable amount of research studies that report mainly an increase in the extracerebral space and delay in the maturity of brain circumvolution in preterm children, which are conditions associated with alterations to the CGM [33].

Tich et al. [34] and Kidokoro et al. [35] demonstrated that increased interhemispheric distance and decreased Biparietal and Bifrontal Diameters are markers of significant motor and cognitive deficiencies in 24-month-old children. Additionally, DGM lesions are often associated with motor planning deficits, affecting movement intensity, rhythm, orientation, and amplitude.

## 5. Conclusions

This study did not find a significant association between the quality of Writhing Movements and motor development. However, a strong association was observed between the presentation of Fidgety Movements and motor development, with 92% of children classified as Absent Fidgety showing abnormal or suspicious motor outcomes.

These results highlight the potential of GM assessment as an early indicator of neurological risk, particularly in identifying infants at higher risk for motor impairment. However, given the limitations of observational studies and small sample sizes, future research should employ standardized imaging protocols and larger cohorts to validate these associations and enhance the clinical utility of GM assessment in early neurodevelopmental screening, especially when the population has no access to technological resources.

Additionally, it would be worth it to carry out a study taking into account other clinical, biological, and sociodemographic variables, such as breastfeeding, race, local or immigrant status, duration of the hospital stay, usage of corticosteroids, usage of surfactants, presence of ventricular hemorrhage, time using a mechanical ventilator, presence of infections, phototherapy, among others.

## Figures and Tables

**Table 1:** Sample characterization: risk factors.

	n	%
**Sex**		
Female	22	44
Male	28	56
**Gestational Age Type** **(Preterm/term-born)**		
Extremely Preterm	5	10
Very Preterm	29	58
Late Preterm	11	22
Term Born	5	10
**Maternal Preeclampsia**		
No	32	64
Yes	18	36
**Gestational Syphilis**		
No	46	92
Yes	4	8
**Bronchopulmonary Dysplasia**		
No	15	30
Yes	35	70
**Respiratory Distress**		
No	1	2
Yes	49	98
**Perinatal Asphyxia**		
No	45	90
Yes	5	10

**Table 2:** Relational analysis between Fidgety Movements and motor development.

AIMS Motor Development Result:	Fidgety Stage Result				*p*=
	F− n = 12		F++ n = 38		
	n	%	n	%	
Without Abnormalities	3	25.0%	37	97.4%	<0.001 *
Abnormal	9	75.0%	1	2.6%	

* Significant association with a 95% confidence (*p*-value < 0.05).

## Data Availability

The data presented in this study are available on request from the corresponding author due to ethical reasons.

## References

[1] Himmelmann K., Ahlin K., Jacobsson B.O., Cans C., Thorsen P. (2011). Risk factors for cerebral palsy in children born at term. Acta Obstet. Gynecol. Scand..

[2] Organización Mundial de la Salud (2017). Estadísticas de Parálisis Cerebral en América—Centro de Cirugía Especial de México, IAP.

[3] Ashwal S., Russman B.S., Blasco P.A., Miller G., Sandler A., Shevell M., Stevenson R. (2004). Practice Parameter: Diagnostic assessment of the child with cerebral palsy. Report of the Quality Standards Subcommittee of the American Academy of Neurology and the Practice Committee of the Child Neurology Society. Neurology.

[4] Aguilar F. (2003). Plasticidad Cerebral. Rev. Médica Del Inst. Mex. Del Seguro Soc..

[5] Van Kranen-Mastenbroek V., van Oostenbrugge R., Palmans L., Stevens A., Kingma H., Blanco C., Hasaart T., Vles J. (1992). Inter- and intra-observer agreement in the assessment of the quality of spontaneous movements in the newborn. Brain Dev..

[6] Einspieler C., Prechtl H.F.R. (2005). Prechtl’s assessment of general movements: A diagnostic tool for the functional assessment of the young nervous system. Ment. Retard. Dev. Disabil. Res. Rev..

[7] Valentin T., Uhl K., Einspieler C. (2005). The effectiveness of training in Prechtl’s method on the qualitative assessment of general movements. Early Hum. Dev..

[8] Bosanquet M., Copeland L., Ware R., Boyd R. (2013). A systematic review of tests to predict cerebral palsy in young children. Dev. Med. Child Neurol..

[9] Tomantschger I., Herrero D., Einspieler C., Hamamura C., Voos M.C., Marschik P.B. (2018). The general movement assessment in non-European low- and middle-income countries. Rev. de Saude Publica.

[10] Peyton C., Einspieler C. (2018). General movements: A behavioral biomarker of later motor and cognitive dysfunction in NICU graduates. Pediatr. Ann..

[11] Hernández R., Fernández C., Baptista P. (2014). Metodología de la Investigación.

[12] Spittle A.J., Anderson P.J., Tapawan S.J., Doyle L.W., Cheong J.L.Y. (2021). Early developmental screening and intervention for high-risk neonates—From research to clinical benefits. Semin. Fetal Neonatal Med..

[13] Aguilar S. (2005). Fórmulas para el cálculo de la muestra en investigaciones de salud. Salud En Tabasco.

[14] Asociación Médica Mundial (2015). Declaración de Helsinki de la AMM—Principios Éticos para las Investigaciones Médicas en Seres Humanos.

[15] Ministerio de Salud y Protección Social de Colombia Resolución Numero 8430 de 1993. https://www.minsalud.gov.co/sites/rid/lists/bibliotecadigital/ride/de/dij/resolucion-8430-de-1993.pdf.

[16] Yildirim C., Asalioğlu A., Coşkun Y., Acar G., Akman I. (2022). General movements assessment and Alberta Infant Motor Scale in neurodevelopmental outcome of preterm infants. Pediatr. Neonatol..

[17] Snider L.M., Majnemer A., Mazer B., Campbell S., Bos A.F. (2008). A comparison of the general movements assessment with traditional approaches to newborn and infant assessment: Concurrent validity. Early Hum. Dev..

[18] Van Iersel P.A., Bakker S.C., Jonker A.J., Hadders-Algra M. (2009). Quality of general movements in term infants with asphyxia. Early Hum. Dev..

[19] Ferrari F., Cioni G., Einspieler C., Roversi M.F., Bos A.F., Paolicelli P.B., Ranzi A., Prechtl H.F.R. (2002). Cramped Synchronized General Movements in Preterm Infants as an Early Marker for Cerebral Palsy. Arch. Pediatr. Adolesc. Med..

[20] Zhussupova Z., Jaxybayeva A., Ayaganov D., Tekebayeva L., Mamedbayli A., Tamadon A., Zharmakhanova G. (2024). General movement assessment efficacy for assessment of nervous system integrity in children after hypoxic-ischemic encephalopathy in middle income countries. Early Hum. Dev..

[21] Wang J., Shen X., Hu X., Yang H., Yin H., Zhu X., Gao H., Wu Y., Meng F. (2021). Early detection relationship of cerebral palsy markers using brain structure and general movements in infants born <32 weeks gestational age. Early Hum. Dev..

[22] Harpster K., Merhar S., Priyanka Illapani V.S., Peyton C., Kline-Fath B., Parikh N.A. (2021). Associations Between Early Structural Magnetic Resonance Imaging, Hammersmith Infant Neurological Examination, and General Movements Assessment in Infants Born Very Preterm. J. Pediatr..

[23] Spittle A.J., Doyle L.W., Anderson P.J., Inder T.E., Lee K.J., Boyd R.N., Cheong J.L.Y. (2010). Reduced cerebellar diameter in very preterm infants with abnormal general movements. Early Hum. Dev..

[24] Peyton C., Yang E., Kocherginsky M., Adde L., Fjørtoft T., Støen R., Bos A.F., Einspieler C., Schreiber M.D., Msall M.E. (2016). Relationship between white matter pathology and performance on the General Movement Assessment and the Test of Infant Motor Performance in very preterm infants. Early Hum. Dev..

[25] Peyton C., Yang E., Msall M.E., Adde L., Støen R., Fjørtoft T., Bos A.F., Einspieler C., Zhou Y., Schreiber M.D. (2017). White Matter Injury and General Movements in High-Risk Preterm Infants. Am. J. Neuroradiol..

[26] Maeda T., Iwata H., Sekiguchi K., Takahashi M., Ihara K. (2019). The association between brain morphological development and the quality of general movements. Brain Dev..

[27] Anderson P.J., Cheong J.L.Y., Thompson D.K. (2015). The predictive validity of neonatal MRI for neurodevelopmental outcome in very preterm children. Semin. Perinatol..

[28] Jiang H., Liu H., Huang T., Wu L., Wu F., Liu C., Wang M., Jin C., Yang J., Li X. (2021). Structural network performance for early diagnosis of spastic cerebral palsy in periventricular white matter injury. Brain Imaging Behav..

[29] Spittle A.J., Brown N.C., Doyle L.W., Boyd R.N., Hunt R.W., Bear M., Inder T.E. (2008). Quality of General Movements Is Related to White Matter Pathology in Very Preterm Infants. Pediatrics.

[30] Olsen J.E., Brown N.C., Eeles A.L., Einspieler C., Lee K.J., Thompson D.K., Anderson P.J., Cheong J.L.Y., Doyle L.W., Spittle A.J. (2016). Early general movements and brain magnetic resonance imaging at term-equivalent age in infants born <30weeks’ gestation. Early Hum. Dev..

[31] Ferrari F., Todeschini A., Guidotti I., Martinez-Biarge M., Roversi M.F., Berardi A., Ranzi A., Cowan F.M., Rutherford M.A. (2011). General Movements in Full-Term Infants with Perinatal Asphyxia Are Related to Basal Ganglia and Thalamic Lesions. J. Pediatr..

[32] Franki I., Mailleux L., Emsell L., Peedima M.-L., Fehrenbach A., Feys H., Ortibus E. (2020). The relationship between neuroimaging and motor outcome in children with cerebral palsy: A systematic review—Part A. Structural imaging. Res. Dev. Disabil..

[33] Kidokoro H., Neil J.J., Inder T.E. (2013). New MR Imaging Assessment Tool to Define Brain Abnormalities in Very Preterm Infants at Term. Am. J. Neuroradiol..

[34] Tich S.N.T., Anderson P.J., Shimony J.S., Hunt R.W., Doyle L.W., Inder T.E. (2009). A Novel Quantitative Simple Brain Metric Using MR Imaging for Preterm Infants. Am. J. Neuroradiol..

[35] Kidokoro H., Anderson P.J., Doyle L.W., Woodward L.J., Neil J.J., Inder T.E. (2014). Brain Injury and Altered Brain Growth in Preterm Infants: Predictors and Prognosis. Pediatrics.

